# Recommendations and Best Practices for the Risk Assessment of Pressure Injuries in Adults Admitted to Intensive Care Units: A Scoping Review

**DOI:** 10.3390/nursrep15040128

**Published:** 2025-04-11

**Authors:** Ricardo Picoito, Tânia Manuel, Sofia Vieira, Rita Azevedo, Elisabete Nunes, Paulo Alves

**Affiliations:** 1 Faculty of Health Sciences and Nursing, Universidade Católica Portuguesa, 1649-023 Lisbon, Portugal; 2 Wounds Research Lab, Center for Interdisciplinary Research in Health|CIIS, 4169-005 Porto, Portugal; tmscarvalho@hotmail.com (T.M.); s-srovieira@ucp.pt (S.V.); s-arrazevedo@ucp.pt (R.A.); pjalves@ucp.pt (P.A.); 3 Núcleo de Investigação e Formação em Enfermagem, Centro de Inovação e Investigação Clínica da Unidade Local de Saúde Lisboa Ocidental, 1449-005 Lisbon, Portugal; 4 Nursing Research Innovation and Development Centre of Lisbon (CIDNUR), Nursing School of Lisbon, 1600-190 Lisbon, Portugal; enunes@esel.pt; 5 Faculty of Health Sciences and Nursing, Universidade Católica Portuguesa, 4169-005 Porto, Portugal; 6 Nursing School of Lisbon, 1990-096 Lisbon, Portugal

**Keywords:** recommendations, statements of good practice, risk assessment, pressure injury, intensive care units

## Abstract

*Background*: The prevention of pressure injuries depends on the early and correct assessment of at-risk patients. Since risk assessment involves more than using a risk factor instrument, we intend to map the existing recommendations and statements of good practice for pressure injury risk assessment in adults admitted to intensive care units, as well as identify the strengths of the evidence and recommendations in the literature. **Methods**: This study is a scoping review, guided by the Joanna Briggs Institute framework. The Preferred Reporting Items for Systematic Reviews and Meta-Analyses Extension for Scoping Reviews was adopted as a guide for writing this study. **Results**: Searches were carried out in six databases, resulting in 794 studies, of which 15 were included. The recommendations and statements of good practice were grouped into five categories: risk assessment instruments, skin assessment, medical device surveillance, other alternatives to risk assessment, and implementing best practices in clinical settings. The strengths of the evidence and recommendations were identified when available in the literature. *Conclusions*: The mapping showed that the evidence is sufficient to indicate recommendations and statements of good practice for the risk assessment of pressure injuries in adults admitted to intensive care units. The protocol was retrospectively registered in the Open Science Framework on the 4th of August of 2023.

## 1. Introduction

The development of pressure injuries (PIs) is a common complication in various healthcare settings, mainly affecting critically ill patients. This phenomenon is considered a relevant adverse effect, associated with an increased risk of hospital complications, morbidity, hospital infections, increased recovery time, and decreased quality of life [1]. Furthermore, it represents a challenge for healthcare institutions due to the increased demands on nursing teams and high costs related to specific products and treatments [2].

The prevention of PIs relies heavily on the early and accurate identification of at-risk patients. A risk assessment is a systematic process that evaluates the interplay between patient-specific factors and environmental conditions to identify potential contributors to tissue damage. This assessment aims to determine modifiable risk factors, assess the feasibility of mitigating these risks, and guide the implementation of targeted preventive or protective interventions [3].

The impact of risk factors on hospitalized patients is substantial, contributing to the development of PIs, especially in critically ill patients admitted to the ICU, reaching a prevalence rate between 16.9 and 23.8% (95% CI) and an incidence rate between 10.0 and 25.9% (95% CI) [4]. An international study of the prevalence of PIs in ICU patients reported that 59% of all injuries identified in a hospital setting were acquired in the ICU [5].

It appears that, in general, in ICUs there is a higher incidence and prevalence rate compared to other areas of the hospital, which may be explained by the various risk factors associated with critically ill patients [6]. This diversity of factors makes it difficult to correctly assess risk due to the specific characteristics of this population, the high degree of dependence of the people hospitalized there, and limitations associated with the environment and its users, such as hemodynamic instability, the restriction of movement for long periods, and the use of sedatives and analgesics that hinder patients’ mobility or limit their sensory perception [7].

Several factors contribute to the development of PIs, but their comprehensive assessment is challenging due to the variability in the severity and impact of these factors. Given this complexity, over the last few decades, researchers have developed risk assessment instruments—generalist and specific—for different care contexts. In ICUs, where multiple risk factors are present, it is essential to use more comprehensive instruments aimed at this context to consider the specificities of critically ill patients, rather than generalist approaches [8,9].

International organizations such as the National Pressure Injury Advisory Panel (NPIAP), in partnership with the European Pressure Ulcer Advisory Panel (EPUAP) and the Pan-Pacific Pressure Injury Alliance (PPPIA), have developed guidelines for the prevention and treatment of PIs [10]. In 2016, these guidelines were updated, replacing the term “pressure ulcer” with “pressure injury”, describing more precisely both intact and ulcerated skin injuries. Pressure injury (PI) is defined as localized damage to the skin and/or underlying soft tissue, usually over a bony prominence or related to the use of a medical device or other artefact. The lesion may appear on intact skin or as an open ulcer; it may be painful and occur as a result of intense pressure in combination with shear force [11].

A comprehensive literature search on methods for the risk assessment of PIs in adults admitted to the hospital revealed international guidelines, supported by studies with a high level of evidence and strength of recommendation [11,12]. These studies support that global risk assessment should be structured and include the following: i. Global clinical assessment, including degree of mobility, urinary/faecal incontinence, changes in sensitivity, change in state of consciousness, vascular disease, and nutritional status; ii. Complete and accurate skin assessment; iii. Use of a risk assessment scale with the evaluation of additional risk factors; iv. Use of clinical judgment in interpreting results. We did not find, in the international literature, specific recommendations for the risk assessment of patients admitted to the ICU that were duly robust and organized.

Recommendations and best practice statements are evidence-based and assist healthcare professionals, clients, and informal caregivers make appropriate decisions in specific clinical contexts [11]. However, their applicability depends on an individualized assessment, patient preferences, and available resources [11].

The strengths of evidence for recommendations and statements of good practice are assigned based on the study design, considering the quantity, level of evidence, and consistency of the results presented [10]. The strength of recommendations is determined by a consensual voting process [10], being an important tool for health professionals to prioritize the interventions to be carried out. The strengths of evidence are categorized as follows [11] (p. 3):

A—More than one high-quality level 1 study providing direct evidence. Consistent body of evidence.

B1—Level 1 study of moderate or low quality, or level 2 study of high or moderate quality, both providing direct evidence. Most studies present consistent results, with inconsistencies being explainable.

B2—Low-quality level 2 study, or level 3 or 4 studies, providing direct evidence. Most studies present consistent results, with inconsistencies being explainable.

C—Level 5 studies (indirect evidence). The body of evidence has inconsistencies that cannot be explained, reflecting genuine uncertainty regarding the topic.

Good practice statements (GPS): Consensus opinions that are not based on a body of evidence, such as those mentioned previously, but that are considered relevant to clinical practice by the Partner Organization Guideline Governance Group.

Strengths of recommendation are also divided into five categories [11] (p. 3):

↑↑ Strong positive recommendation: application strongly advised.
↑ Weak positive recommendation: possible application.
↔ No specific recommendation: there is insufficient evidence to recommend or advise against.
↓ Weak negative recommendation: better not to apply.
↓↓ Strong negative recommendation: application strongly advised against.

This system facilitates the interpretation and application of guidelines, promoting informed decisions and evidence-based practice. The adoption of evidence-based guidelines is essential to reduce the risk of PIs, promoting better clinical outcomes.

A preliminary literature search indicated a gap in systematic reviews addressing PI risk assessment strategies in ICU patients. While extensive research focuses on PI prevention, there is a limited synthesis of specific risk assessment recommendations and their strengths of evidence. To address this gap, this scoping review aims to systematically map existing recommendations and statements of good practice for PI risk assessment in ICU patients and evaluate the level of evidence supporting these recommendations. By categorizing and analysing the available guidance, this study provides a comprehensive reference for clinical decision-making and future research.

## 2. Materials and Methods

### 2.1. Type of Study

This scoping review aimed to systematically map recommendations and best practice statements for the risk assessment of PIs in adults admitted to ICUs, while also evaluating the strength of evidence and the robustness of the recommendations. The methodology was guided by the Joanna Briggs Institute (JBI) framework, ensuring rigor and adherence to best practices in scoping reviews [13]. The study was reported in accordance with the Preferred Reporting Items for Systematic Reviews and Meta-Analyses Extension for Scoping Reviews (PRISMA-ScR) guidelines [14]. Furthermore, the protocol for this review was prospectively registered in the Open Science Framework under the following DOI: 10.17605/OSF.IO/5NF3U.

This review was conducted through a comprehensive search of multiple databases, including CINAHL Complete via EBSCOhost, MEDLINE Complete via EBSCOhost, PubMed, the Cochrane Database of Systematic Reviews, SciELO, and the EBSCO Discovery Service. The search strategy was developed using a combination of controlled vocabulary and free-text terms, incorporating descriptors validated by CINAHL Subject Headings and Medical Subject Headings from MEDLINE, alongside carefully selected keywords to ensure comprehensive coverage of the relevant literature.

No time limit was defined, considering the absence of previous studies on the topic in the international literature. The survey was carried out in February 2025. Articles from different types of research were included in the languages dominated by the researchers: English, Portuguese, and Spanish. Letters to the editor, event annal summaries, and articles that did not present information related to the population, concept, and context of the study were excluded.

Studies were included that addressed recommendations and statements of good practice for the risk assessment of PI in adults admitted to the ICU, regardless of the pathology or cause of hospitalization. The study’s initial sample included 794 articles found in databases and the grey literature available on Google Scholar. The selection of studies was carried out by three independent reviewers, based on the title, indexed terms, summary, and in the last phase, complete reading. After data extraction, differences that arose between reviewers were resolved through discussion until consensus was reached.

The extracted data were recorded in an information collection instrument adapted from the model recommended by JBI and organized in a data table in Microsoft Word 2016 [13].

### 2.2. Data Collection

Data collection was conducted using the PCC (Participants, Concept, and Context) mnemonic framework to formulate the research question. The participants included adults in critical condition, while the concept focused on recommendations and best practice statements for assessing PI risk, along with the strength of evidence and recommendations. The context encompassed admission to the ICU across various specialties, including multipurpose, medical, surgical, and trauma. The research question guiding this study was as follows: “*What are the Recommendations and Statements of Good Practice for Risk Assessment of PI in Adults Admitted to Intensive Care Units?*”

Based on this question, a structured search strategy was developed, utilizing a combination of controlled vocabulary and keywords, which were systematically applied across multiple databases. Advanced search techniques were employed to ensure the comprehensive retrieval of the relevant literature. Published articles, reviews, and other pertinent documents were included in the analysis. The data collection process was carried out in three distinct stages to ensure methodological rigor and completeness:

1—Initial search: floating search in the CINAHL and MEDLINE databases via EBSCOhost; analysing the articles by the words in the title, abstract, and indexed terms used, and identifying relevant descriptors and keywords.

2—Advanced and complementary search: after defining the descriptors, a new structured search was carried out in the defined databases based on defined criteria, as shown in Table 1.

As a complement, another search was carried out using additional sources (Google Scholar), adding six studies.

3—Document analysis: in the third stage, the bibliographic references of the selected studies were analysed according to the pre-established criteria, and no new studies were included.

### 2.3. Data Extraction

The extracted data were organized in a table containing detailed information about the article title, year of publication, newspaper, country, and study design in descending chronological order. Only articles that met the inclusion criteria were included, covering experimental and observational studies, systematic reviews, and reports from international organizations.

#### 2.3.1. Data Treatment and Analysis

Data were analysed in accordance with the review objectives using content analysis. The results are presented in descriptive tables that highlight relevant information, according to the objectives of the scoping review. Complementing the tables, a descriptive narrative text was developed to contextualize and interpret the data presented.

#### 2.3.2. Ethical Aspects

During the preparation of the article, ethical principles were strictly respected, including the identification of the authors, whose work was used as a scientific basis. All references were duly cited, ensuring justice and due credit to the intellectual property of the consulted authors.

## 3. Results

**Figure 1:**
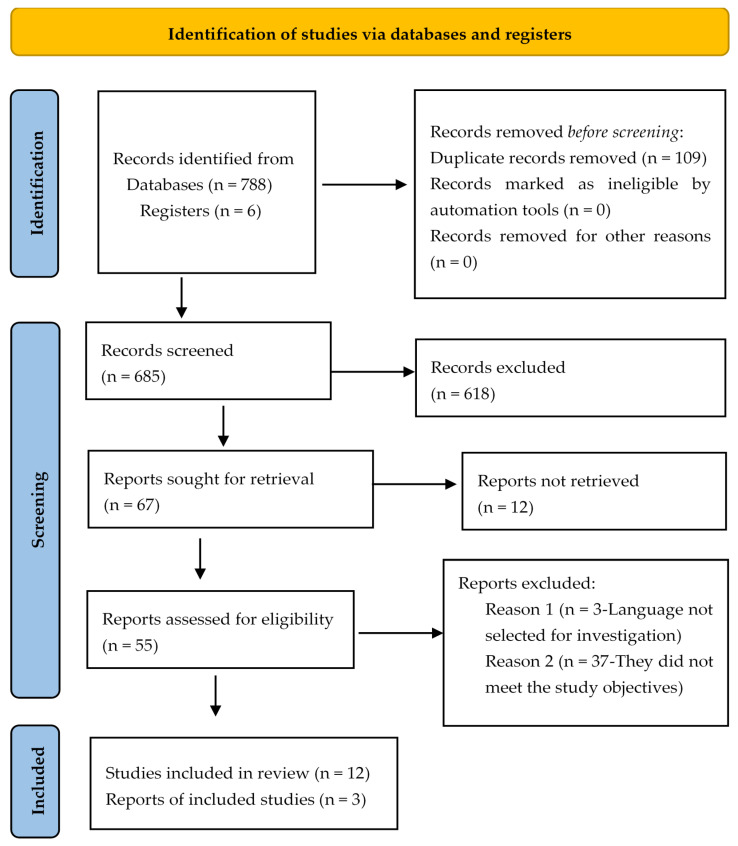
Flowchart of the study selection process adapted from PRISMA-ScR [14].

In the search carried out, 794 potentially eligible studies were identified in the databases, 109 duplicate articles were removed, and 618 studies were excluded after reading the title, indexed terms, and abstract. Of the 67 articles remaining for evaluation, 12 articles were excluded for not having the full or accessible text available in the databases; 3 articles because they were not in the languages selected for the research; and 37 articles were eliminated because they did not meet the study objectives. As a result, the final review sample consisted of 15 articles, as shown in Figure 1.

Table 2 presents the characterization of the 15 studies included in the review, detailing information such as year and country of publication, article title, study design, aims, and key conclusions. This description allows an overview of the origin, context, and design of the selected studies, contributing to the critical analysis and understanding of the available evidence.

The analysis of the selected studies revealed a wide geographic coverage, covering almost all continents. In terms of distribution, six studies were European [12,15,16,17,19,25], four American [21,22,24,27], and three Asian [18,20,23], with one study from Oceania [26]. We also highlight an article involving joint contributions from European, American, and Pan-Pacific entities [11]. In terms of nationalities, three publications from the United Kingdom [12,16,19] and Brazil [21,22,24] stand out. Other countries represented with one publication each were Turkey [15], Italy [17], Saudi Arabia [18], Indonesia [20], Thailand [23], Ireland [25], Australia [26], and the United States of America [27].

The first study included dates back to 2014 [12], highlighting a growing concern about the topic over the years. In the past four years, 9 of the 15 included studies were published, representing 60% of the sample, reflecting a significant increase in interest and research on the topic.

Regarding study design, eight original research studies were considered for inclusion, five of which were experimental and three observational. Four literature review articles and three reports were also included.

Based on the results extracted from the studies, the recommendations and statements of good practice were grouped into five broad categories, as detailed in Table 3: risk assessment instruments, skin assessment, medical device surveillance, other alternatives for risk assessment, and implementing best practices in clinical settings.

In addition to the recommendations and statements of good practice, the strengths of evidence and strengths of recommendation available for each recommendation or statement are also presented in Table 3.

### 3.1. Risk Assessment Instruments

The use of PI risk assessment instruments is widely recommended, being reported in at least six articles [11,12,15,17,18,24]. Healthcare professionals perform a structured and comprehensive risk assessment using a validated and sufficient scale, specific to the ICU environment [11,12,15,17]. This assessment should also consider additional risk factors, both modifiable and non-modifiable, that may not be included in the risk assessment tool using a (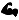 GPS) [11]. The assessment must be carried out upon admission or within the first 8 h, and then daily [11,15]. Whenever there is any significant change in the patient’s clinical condition (after surgery, worsening of an underlying condition, or change in mobility) [11,12,15], the healthcare professional must carry out a new assessment (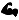 GPS).

Several risk assessment instruments are mentioned in the literature, including generalist options, where the Braden scale clearly stands out [11,17,20,24]. Other specific ones include the CALCULATE scale [16,17]; Cubbin–Jackson scale [11,17,20,24]; COMHON index [11,17]; EVARUCI scale [11,17,20,24], and the Suriadi and Sanada scale [11,20,24]. However, the literature does not clearly identify which of these instruments performs best in terms of predictive validity. Although scales are valuable tools, the results must be interpreted together with clinical judgment. This should prevail over scales when there are discrepancies, ensuring a more personalized and effective approach for each patient [11,12] (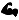 GPS).

This integrated approach aims to optimize risk identification and management, promoting better outcomes in the care provided.

### 3.2. Skin Assessment

A rigorous and comprehensive assessment of the skin and soft tissues from head to toe [11,12,15,16,26], with a focus on the skin covering bony prominences, is widely recognised and recommended by health professionals (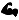 GPS; expert opinion).

- Recommendations for a complete and correct assessment include the following:

1—Vascular/perfusion assessment:

Examine lower limbs, heels, and feet. 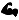 B2; ↑↑ [11].

2—Skin assessment characteristics:

Observe heat, colour, turgor, humidity, edema, and erythema. 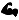 A; ↑↑ [11,12,15].

Examine signs of maceration, especially in skin folds, with extra attention in obese patients. 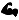 expert opinion [11].

3—Temperature of the skin and soft tissues:

Evaluate temperature variations. 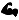 B1; ↑ [11].

4—Identification of erythema:

Differentiate between clearing and non-clearing erythema using digital pressure or a clear disc. Assess the extent of the erythema. 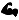 B1; ↑↑ [11,12].

5—Assessment under prophylactic dressings:

Check the integrity of the skin beneath dressings. 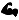 expert opinion [11].

6—Consider the patient’s pain or discomfort:

Incorporate reports of pain as part of the assessment. 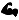 not available (n/a) [12].

7—Moments of assessment:

Admission or within the first 8 h, and thereafter every 8 h. 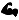 GPS [11,15].

Assess before discharge from service. 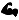 GPS [11].

Increase frequency in case of clinical deterioration. 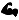 expert opinion [11].

8—Perfusion and formalized risk assessment:

Combining tissue perfusion with validated risk assessment tools. 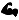 n/a [11].

- The InSPiRE bundle was developed to prevent PIs [26], and highlights the complete assessment of skin integrity. It is recommended that this assessment be performed within the first 4 h after admission to the ICU, then repeated and documented every 12 h. Complete documentation in the clinical information system involves a drop-down menu of standardized descriptors, such as skin colour, moisture, texture, edema, and turgor [26]. 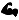 n/a.
- Technological alternatives to evaluate the skin.

The Sub-Epidermal Moister (SEM) Scanner [11,16,19,25] is a portable, wireless, non-invasive device approved by CE (class IIa) and the FDA (DEN170021, 2017) that assesses sub-epidermal moisture, an early indicator of pressure-induced damage that occurs before visible signs appear on the skin or tissue. This detects early-stage and deep PIs by evaluating the anatomical areas of highest risk [19]. When used alongside routine clinical care, this provides crucial information about incipient pressure-induced damage, enabling early preventative interventions before the onset of PIs [11,19]. 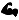 B2; ↔.

Thermography measures the proportion of the spectrum of infrared energy released by the body’s skin and portrays it in an image where different colours are equivalent to the variation in skin temperature [25]. 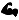 n/a.

Ultrasound is a technique that emits sound waves to create soft tissue images capable of detecting tissue damage and has emerged as a promising non-invasive technology for the early detection of deep tissue damage, enabling identification before visible signs manifest on the skin [25]. 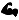 n/a.

These technological tools complement clinical assessment, contributing to a more accurate and early approach in preventing pressure injuries.

### 3.3. Medical Device Surveillance

Medical devices are essential tools for providing care and are increasingly recognised as common and accepted practices, particularly in ICU environments. When evaluating the risk of medical-device-related pressure injury (MDRPI) development, it is crucial to acknowledge that all patients with a medical device are at risk, and the likelihood of developing such injuries increases with the number of devices used [16,19,21,22].

- In the presence of medical devices, the following steps are recommended:

1—Assessment of Skin Associated with Medical Devices:

Assess the skin around and under medical devices every 12 h. 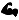 expert opinion [11,15,16,21,22].

Carry out a systematic assessment of the pressure exerted by the device, paying special attention to areas in direct contact. 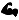 expert opinion [11,21].

Perform a head-to-toe assessment, ensuring a complete and detailed analysis. 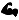 expert opinion [11,21].

2—Incorporation into Daily Practice:

Make assessment a daily routine, with increased frequency for high-risk devices or in patients with systemic conditions, such as hypoalbuminemia. 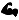 GPS [11,16,21].

3—Device Tension Monitoring:

Regularly check the tension of medical device fixings to avoid excessive pressure.

Whenever possible, ask the patient to self-assess their comfort. 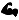 C; ↑ [11].

4—Conditions of the Skin and Underlying Tissues:

Consider characteristics such as scars from previous injuries, local atrophy, and edema.

Regularly reassess the clinical need to maintain the device. 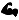 expert opinion [16].

5—Clinical Judgment:

Adjust the frequency of assessment based on the patient’s clinical condition and the risk associated with the device. 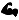 expert opinion [16].

- The growing concern about this issue motivated the creation of preventive strategies, such as SKINCARE [18], a bundle of interventions developed internationally. This set of interventions includes a key intervention in risk assessment: the inspection of the skin under medical devices at least twice a day (intervention I). High-risk patients will require more frequent assessments [18]. 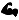 n/a.

### 3.4. Other Alternatives to Risk Assessment

The use of new models and technologies appears to contribute to the risk assessment of PIs. Although the international literature presents some alternatives, it does not yet present the strengths of recommendation and evidence for each intervention. The following were identified:

- CAVE—cardiovascular–low albumin–ventilator–edema—is a simple predictive risk scoring model that includes four scored factors, namely the presence of cardiovascular disease = 2; + the presence of serum albumin < 3.3 mg/dL = 2; + the presence of mechanical ventilation = 1.5; and + the presence of edema = 1 [23]. 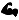 n/a.
- The calculation of body mass index (BMI) appeared to be associated with the occurrence of PIs in ICU patients [27]. 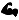 n/a.
- The multi-pad pressure evaluator [24] is an instrument that measures the pressure exerted on bone prominences, applicable in different locations on the skin to quantify the intensity of pressure [28]. 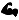 n/a.

### 3.5. Implementing Best Practices in Clinical Settings

The application of organizational and educational strategies is essential for reducing the incidence of PIs in clinical settings. The following recommendations highlight priority actions:

- Evaluation and Maximization of Resources:

Assess the availability and quality of equipment, ensuring that standards for its use are appropriate. This analysis should be integrated into a quality improvement plan, aiming to reduce the incidence of PIs [11]. 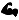 B1; ↑↑.

- Clinical Decision Support Tools:

Implement and provide clinical decision support tools as an integral part of organizational quality programs. These tools help professionals make evidence-based decisions when managing PI [11]. 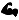 B1; ↑↑.

- Assessment and Training of Health Professionals:

Perform a comprehensive assessment of professionals’ knowledge of PIs. This analysis allows the identification of gaps and facilitates the implementation of continuing education programs, in addition to strengthening initiatives to improve the quality of care provided [11]. 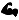 B1; ↑↑.

## 4. Discussion

The results of this review highlighted a wide variety of recommendations related to PI risk assessment, with differing strengths of recommendation and evidence. These recommendations were systematically grouped into five main categories: risk assessment instruments, skin assessment, medical device surveillance, other alternatives for risk assessment, and implementing best practices in clinical settings.

Categorizing recommendations allows us to identify priority areas for intervention and guide clinical practice based on the integration of robust evidence and organizational strategies. This holistic approach is essential for preventing PIs, especially in the ICU, where patients are more vulnerable.

### 4.1. Risk Assessment Instruments

The use of PI risk assessment instruments is widely recognized as standard practice; these should be a “crutch” for the health professional and never prevail in relation to their clinical judgment [11,12].

The literature unanimously indicates that risk assessment should occur immediately upon admission, ideally within the first 8 h, and be repeated daily [11,15]. This assessment must be structured and comprehensive, supported by validated scales sufficiently specific for the ICU environment [11,12,15,17]. However, choosing the ideal scale presents challenges, considering the diversity of available instruments and their limitations in different contexts.

Studies demonstrate that generalist scales presented lower sensitivity and specificity values than specific scales. This evidence reinforces the need to prioritize the use and dissemination of specific scales due to their better predictive capacity in critical settings [24].

Among the generalist scales, the Braden scale stands out, identified in four studies [11,17,20,24], being one of the most used and validated scales in different care contexts. Despite being the most tested scale in ICUs [24,29], a meta-analysis of 11 studies involving 10,044 participants revealed that its predictive capacity is moderate and insufficient to exclude the high risk of developing PIs in the ICU [30]. Furthermore, the accuracy’s scale is significantly reduced in ventilated, dialyzed, surgical patients and those using inotropic drugs [31]. A recent study assessed the inter-rater reliability of the Braden scale and its subscales, concluding that it lacks sufficient reliability for use in ICU settings. The study discourages its application in the ICU and emphasizes the need for alternative, context-specific assessment tools that better address the risk factors of critically ill patients [32].

The modification of the Braden scale, replacing the nutrition subscale with serum albumin, resulted in the Braden ALB scale [17], which demonstrated promising validity and reliability values [33]. In a prospective study [34] that compared four risk assessment scales, the Braden ALB showed better predictive performance than the CALCULATE scale, Braden scale, and COMHON index. The Braden ALB scale showed sufficient sensitivity and specificity values for use in ICUs, being potentially preferable to the original version [17]. However, further studies are needed to strengthen predictive validity data.

One study identified the Norton scale, which showed accuracy values comparable to the Braden scale [17]. However, only two prospective studies assessing predictive validity were found in the international literature, reporting moderate-to-low precision values [35]. Based on these results, the Norton scale is not recommended for use in the ICU.

Among the specific scales, the Cubbin–Jackson scale was identified in four studies [11,17,20,24], presenting more comprehensive risk assessment aspects for critically ill patients [20], with sensitivity and specificity values suitable for use in the ICU. This scale may be preferred to the Braden scale [17]. Cubbin-Jackson reflects the complex condition of critically ill patients, addressing factors such as comorbidities, unstable hemodynamic status, the use of vasoactive medication, and mechanical ventilation. Furthermore, it stands out for its ability to monitor rapid changes in the clinical condition of critically ill patients, making it a more sustainable and adjusted tool for this context [20].

Four studies identified the EVARUCI scale, a specific scale for use in the ICU [11,17,20,24]. Composed of five domains—consciousness, hemodynamic status, respiratory status, mobility, and others—EVARUCI has demonstrated effectiveness in tracking PIs in ICU patients due to its high predictive value [20,24], making it a promising option for use in the ICU.

The Suriadi and Sanada scale, identified in three studies [11,20,24], evaluates three main domains: interface pressure, temperature, and smoking history. This instrument showed good predictive validity values, effectively detecting PIs in ICU patients [20,24]. However, its use in just two ICUs in Indonesia restricts the generalization of results and highlights the need for additional studies to confirm its robustness in different contexts.

The COMHON index [11,17] and the CALCULATE scale [16,17], specific instruments for ICU risk assessment, were identified in two studies. However, the application of the COMHON index as a tool for risk assessment in ICUs is still not a consensus. A prospective study [34], which compared four risk assessment scales for PIs in critically ill patients, showed that the COMHON index presented the worst performance. To improve its accuracy, it is suggested replacing the nutritional subscale with serum albumin, as this appears to be a more sensitive predictor than the feeding route [33].

The CALCULATE scale, a recent instrument developed specifically for the ICU context [36], demonstrated sensitivity and specificity values suitable for use in critically ill patients, being considered a potentially more effective alternative than the Braden scale [17]. More recently, a prospective cohort study that compared the accuracy of the CALCULATE and Braden scales found that CALCULATE may be more accurate in identifying the risk of developing PIs [37]. Another aspect to be taken into consideration is that when contemplating mechanical ventilation, covering devices such as non-invasive ventilation masks, it takes into account some risk aspects associated with the use of medical devices [16]. Thus, CALCULATE emerges as a promising tool, combining ease of use and greater accuracy in identifying patients at high risk of PI [36].

Selecting the most appropriate risk assessment instrument for ICUs requires evidence from studies evaluating the predictive validity, performance indicators, and user experience of available tools. A scoping review [38] aimed at mapping PI risk assessment instruments in the ICU, as well as assessing their performance and user feedback, identified the EVARUCI and CALCULATE scales as the most effective. Additionally, a systematic review with a meta-analysis [35] that examined the effectiveness of PI risk assessment tools in critically ill patients highlighted four primary instruments: Cubbin–Jackson, EVARUCI, Waterlow, and CALCULATE. These scales demonstrated the highest suitability for PI risk assessment in ICU patients, based on key performance metrics such as sensitivity, specificity, and clinical applicability.

Although all of these scales have strengths, the final choice must consider the specific characteristics of the ICU, the available resources, and the conditions of the patients evaluated. To guarantee their effective application in different settings, additional studies must be invested in to confirm and expand the predictive validity of the most promising scales.

### 4.2. Skin Assessment

Rigorous and comprehensive skin and soft tissue assessment from head to toe [11,12,15,26], focusing on areas over bony prominences, is an essential measure, not only in the early identification of PIs, but also in the assessment of the risk of developing them. The condition of the skin and underlying tissue can be an early indicator of changes that precede the development of these lesions, allowing for preventative interventions and timely treatment. This type of assessment, performed routinely, offers a valuable opportunity to detect skin changes, particularly those associated with PIs [11].

The skin and underlying tissue assessment involves a series of recommendations that assist in conducting a correct and comprehensive evaluation. This assessment should be performed by qualified health professionals, utilizing direct observation and clinical judgement, as there is insufficient robust evidence to demonstrate the effectiveness of formal tools or scales for assessing the skin and soft tissues [11].

The observation of skin changes, such as the presence of non-blanchable erythema, has been identified as a predictor for the development of PIs. In a large prognostic study (n = 698), the presence of erythema was linked to more than a twofold increase in the risk of developing Category 2 or greater PI [39]. Localised heat, oedema, and changes in tissue consistency have all been recognised as warning signs for the development of PIs [40]. The early identification of alterations in the colour, temperature, and consistency of skin and tissue enables the implementation of an appropriate prevention and treatment plan.

The integration of intervention bundles into care practice has been increasingly adopted in healthcare institutions as an effective strategy to improve the quality of care provided and prevent PIs. The Institute for Healthcare Improvement encourages the use of scientific evidence-based guidelines to support these practices while promoting patient safety and well-being [41,42]. A notable example is the InSPiRE bundle, which was evaluated in an interventional study conducted in Australia [26]. The results showed that the group that received care following the InSPiRE protocol had a lower cumulative incidence of PIs and a reduction in the severity of these injuries compared to the control group. The systematic and ongoing assessment of patients’ skin and risk of PI, as well as the implementation of personalized prevention measures, are fundamental to preventing PIs [26].

In addition to direct observation of the skin, innovative technologies have been developed to complement PI risk assessment, offering greater accuracy and the early detection of tissue damage. Among these technologies, the SEM scanner and ultrasound stand out as promising auxiliary tools in clinical practice. A study with 284 participants [43] demonstrated that the use of the SEM scanner reduced PIs by 93%. Another study [19], which aimed to measure the impact of adding the SEM scanner to the standard of care, reported a reduction in PI incidence of 81%. However, applying this technology can be challenging in specific populations, such as postoperative, immobilized, or confused patients, due to the need for additional support to perform the examination, which can require more time and resources [19].

Research has investigated the effectiveness of both low- and high-frequency ultrasound in the early diagnosis of PIs [44]. Findings indicate that low-frequency ultrasound demonstrates high sensitivity, specificity, and accuracy in detecting deep tissue damage. In contrast, high-frequency ultrasound exhibited a low-to-moderate correlation between detected tissue changes and PI risk classification. However, the limited number of PI events in this study constrained the ability to fully assess its predictive efficacy [45].

Both the SEM scanner and ultrasound show potential as effective methods for the early prediction of tissue damage and the risk of PIs [25]. Despite promising results, more studies are still needed to consolidate the evidence, determine the cost–benefit, and explore the applicability of these technologies in different populations and clinical contexts [25]. Combining these technologies with traditional assessments could represent a significant advance in preventing PIs, improving patient care and outcomes.

Another alternative method for skin assessment is infrared thermography. The prognostic value of infrared thermography was investigated in a prospective study [46], suggesting that this method was successful in identifying areas with deep tissue injury. Despite promising results, the study had wide confidence intervals, and participants were predominantly Caucasian, limiting the generalizability of the results to other populations. In a prospective cohort study [47], it was found that infrared thermography is able to objectively and accurately identify signs of local hypothermia associated with PIs before they are visible via clinical examination. This method can therefore provide a reliable and timely means of risk assessment, assisting nurses in the early detection of skin changes [47].

Although existing studies suggest that infrared thermography is an effective complementary tool for assessing PI risk, further research is required to validate its applicability across diverse populations and clinical settings. Nevertheless, thermography presents a promising approach, particularly for the early detection of skin and tissue alterations that may not be identified through conventional assessment methods.

### 4.3. Medical Device Surveillance

Assessing a patient’s risk of developing an MDRPI is an essential step in preventing it, as with any PI. Expert guidance and best practice statements highlight the importance of a comprehensive risk assessment that takes into account not only the general risk factors for PIs, but also the additional risk associated with the use of medical devices [16].

Performing frequent skin assessments is considered good practice, although there is no high-quality scientific evidence to support this practice in preventing MDRPIs [11]. Regular skin assessment allows early detection of MDRPIs, enabling rapid and effective interventions. This process should be used to guide patient care planning, ensuring that preventative strategies are incorporated to reduce the risk of PIs, including MDRPIs. The care plan must be dynamic, continually adapting based on the identified risks and the evolution of the patient’s condition [16]. As part of a comprehensive risk assessment, it is also essential to routinely evaluate the continued necessity of medical devices, as prolonged use may increase the risk of MDRPIs. Identifying early signs of pressure-related damage caused by devices should inform clinical decision-making, including adjustments to minimize sustained pressure. For example, the frequent monitoring of tissue integrity around oximetry sensors or endotracheal tubes may allow the early detection of MDRPIs, prompting timely modifications in device positioning or use. These assessments contribute to a proactive approach to identifying at-risk patients and guiding necessary interventions to mitigate further complications [11].

Studies indicate that the greater the number of devices installed, the greater the likelihood of injury formation [21,22]. In a review study [18], patients who developed MDRPIs had, on average, between six and eight devices, a common scenario in critically ill patients. The most aggressive devices include the endotracheal tube and the nasogastric tube [22]. In addition to these, other medical devices frequently associated with skin injuries include respiratory devices, feeding devices, orthopaedic devices, probes, oximeters, cervical collars, patches, and nasogastric tubes [22]. The continuous pressure exerted by these devices on the skin and underlying tissues, especially in patients with limited mobility, considerably increases the risk of tissue damage.

In an exploratory descriptive study carried out in a hospital in Australia [48], the overall incidence of MDRPIs was 27.9%, with the majority (68%) occurring in the ICU. In global terms, the general prevalence of MDRPIs in different hospital sectors is around 7.2% [49]. However, in ICUs, these numbers are significantly higher, ranging from 19.8%, in a study [50] with 253 participants, to 40% in a study [51] carried out in five Turkish ICUs, involving 175 participants.

Given the impact of MDRPIs, it is essential to develop specific, broadly applicable risk assessment tools that consider the additional risks posed by medical devices. These tools should be used routinely and complemented with detailed information about the device used and an individualized clinical assessment [15].

An example of an effective approach is the SKINCARE bundle, which considers essential prevention strategies, such as clinical nursing assessment and documentation, hygiene measures, repositioning, and emerging therapies aimed at preventing MDRPIs in ICU patients [18]. The assumption of efficacy of the SKINCARE bundle in reducing MDRPIs was confirmed by a greater than 90% reduction in total cumulative incidence [18]. Systematic skin assessment remains an essential practice, allowing the early identification of skin damage, enabling interventions at early stages, before lesions worsen. This proactive approach, associated with the use of specific tools and well-structured bundles, is essential to reduce the occurrence of MDRPIs and improve clinical outcomes in vulnerable populations, such as ICU patients.

### 4.4. Other Alternatives for Risk Assessment

Alternative methods have been developed to assess PI risk, offering potential improvements over traditional assessment tools. One such approach is the CAVE predictive model, which demonstrated an Area Under the Receiver Operating Characteristic Curve (AUC-ROC) of 0.80 [23]. However, its overall predictive performance was found to be weak (AUC 0.67) and particularly limited in older patients (AUC 0.57). In contrast, the model performed more effectively in patients under 60 years of age, achieving an AUC of 0.78. Despite its limited predictive validity, the CAVE model is comparable to existing tools used in Thailand and exhibits favourable diagnostic properties for younger patients, suggesting its potential as an alternative screening tool in ICU settings [23]. However, no further studies were found in the international literature investigating this model.

Another method associated with the risk of PIs is BMI. In a retrospective cohort study, it was observed that underweight and extremely obese patients were at greater risk of developing PIs than normal-weight or obese patients. These findings suggest that BMI should be considered a risk factor when evaluating patients [27]. However, the study data are limited and based on just one study [27].

The integration of the multi-pad pressure evaluator instrument [24] also showed promise as an alternative to assess the risk of PIs. A study developing a multi-pad pressure evaluator showed satisfactory reliability and clinical validity [28]. Additionally, a prospective cohort study, which compared its usefulness with the Braden scale, showed that the multi-pad pressure evaluator provided the best balance between sensitivity and specificity, presenting good predictive validity values. The authors suggest that this instrument may be better suited for PI risk assessment in the ICU, making it a viable tool for improving assessment accuracy in complex clinical settings [52].

These alternative approaches reinforce the importance of developing and validating new methods for PI risk assessment, especially in populations with specific characteristics.

### 4.5. Implementing Best Practices in Clinical Settings

Over the past two decades, research into PI prevention and management has grown exponentially, propelled by advancements in evidence-based practice, healthcare policy, and quality improvement initiatives. This progress mirrors the collective efforts of policymakers, educators, and healthcare administrators to embed best practices into clinical care. Evidence indicates that a sustained institutional commitment to quality improvement is directly linked to a lower incidence of PIs [53,54].

The use of algorithms, tools, and clinical decision support protocols, aligned with evidence-based guidelines, has been essential to assist health professionals in selecting appropriate care strategies and equipment for the prevention and treatment of PIs. Multifaceted quality improvement programs that integrate decision support tools for risk assessments have been associated with significant reductions in the occurrence of PIs [55,56]. An example of this is the study conducted by Beeckman et al. [56], which evaluated the effectiveness of an electronic decision support system in creating personalized prevention programs for each individual. The results revealed a significant reduction in category 1 to 4 PIs between the intervention and control groups (7.1% versus 14.6%, *p* < 0.05). Another innovative example was the development of a mobile application to assess, treat, and prevent pressure injuries, called Pressure Injury-App [57]. This application and other findings highlight the importance of integrating technological systems to support clinical decision-making and promote an organizational culture committed to continuous quality improvement. Implementing structured, evidence-based programs can optimize the prevention and treatment of PIs, improving clinical outcomes and promoting safer, more effective care.

Workforce characteristics, including skill mix, staffing levels, and workforce tenure, are critical determinants in the prevention and management of PIs. Evidence suggests that factors such as the number of care hours provided by qualified nurses and the stability of clinical teams influence the successful implementation of best practices [58,59]. Insufficient staffing, high turnover rates, and variability in skill levels have been identified as potential barriers to adherence to evidence-based PI prevention strategies. Consequently, challenges in implementing these practices are associated with a significantly increased risk of PI development.

Assessing healthcare professionals’ knowledge about the prevention and treatment of PIs is essential to guide the implementation of continued education programs and quality improvement initiatives. In a study carried out by Price et al. [60], a pre-intervention survey was applied to assess professionals’ knowledge about PIs. The results were used to develop a multifaceted educational program tailored to identify needs. Following the training, there was a significant increase in the knowledge and competence of healthcare professionals, and a significant reduction in the incidence of PIs [60].

Optimizing human resources is essential for PI risk assessment, but it is not sufficient on its own. Undertaking an assessment of available equipment, such as support surfaces, medical devices, and wound care products, and purchasing them as part of quality improvement initiatives, has been associated with a significant reduction in the incidence of PIs [56,61]. Beeckman et al. [56] integrated an assessment of the quantity and quality of preventive resources into a multifaceted approach, resulting in a reduction in the prevalence of PIs in elderly care facilities (7.1% versus 14.6%).

To facilitate regular and effective risk assessments, healthcare organizations must implement structured policies grounded in best practice recommendations and evidence-based guidelines. These policies should mandate the use of validated risk assessment tools, comprehensive skin and tissue evaluations tailored to the clinical context, and the systematic consideration of medical devices as potential risk factors. Additionally, adherence to scientifically supported protocols ensures consistency in assessment practices. An integrated, multidisciplinary approach aligned with best practices is essential to enhance the early identification of at-risk patients and optimize PI prevention strategies [11].

### 4.6. Limitations

An important limitation was identified: the scarcity of studies in the international literature that directly address recommendations and statements of good practice for assessing the risk of PIs. To overcome this limitation, deepening the topic of prevention, where risk assessment represents the first step, was the viable solution. Furthermore, it is recognized that carrying out more comprehensive research in terms of languages can increase the quantity and quality of available evidence. Some of the recommendations found could have been further explored if there was more literature on the subject. It would also have been significant to identify the strengths of recommendation for all findings. We found no further data in the international literature.

Another limitation of this study is its qualitative nature, which precludes statistical comparisons or meta-analyses of the effectiveness of different risk assessment practices. While scoping reviews provide valuable insights into the existing literature, they do not establish causal relationships or measure intervention effectiveness.

Future research should build upon this work by conducting systematic reviews or meta-analyses to assess the impact of specific recommendations in improving patient outcomes. Detailed exploration of recommendations and good practice statements, integrating updated scientific evidence, can significantly contribute to improving risk assessment strategies and, consequently, PI prevention.

These recommendations are intended to serve as a guide for nurses’ daily practice, helping them to carry out a more appropriate and targeted assessment of the risk of pressure injuries for adults admitted to the ICU. This assessment will allow the correct and effective allocation of human and material resources for the prevention of pressure injuries.

## 5. Conclusions

This scoping review identified a range of recommendations and best practice statements for PI risk assessment in adults admitted to the ICU. These recommendations encompass guidance supported by varying levels of scientific evidence, ranging from expert opinion to more robust empirical findings.

Risk assessment instruments primarily rely on good practice statements rather than high-level scientific evidence. Recommendations focus on the timing and method of application rather than identifying the most appropriate instrument, as the literature does not provide a clear consensus on the most effective tool for ICU settings. The application of risk assessment instruments will only be successfully achieved through the systematic observation of the skin and soft tissues, which is essential for both the early identification of PIs and risk assessments. The evidence supporting skin observation ranges from expert opinion to higher levels of scientific validation.

New risk assessment tools, such as the CAVE model, BMI-based risk stratification, and multi-pad pressure evaluator assessment technologies, show promise in PI risk evaluation. However, additional research is needed to establish their validity and integrate them into evidence-based recommendations with stronger empirical support.

Medical devices are a critical factor in PI risk, particularly in the ICU, where their use is prevalent. Expert consensus emphasizes that a comprehensive skin assessment, with particular attention to device contact areas, is essential for identifying risk. The evidence suggests that the greater the number of medical devices, the higher the likelihood of PI development.

Implementing best practices is fundamental to reducing PI risk, yet institutional limitations—such as shortages in human, physical, and material resources—significantly increase the likelihood of PI development. To mitigate these risks, healthcare institutions must optimize resource utilization, integrate clinical decision-support tools, and invest in continuous staff training. 

While numerous recommendations and best practices for PI risk assessment in ICU patients are widely accepted, the scientific evidence supporting many of them remains limited. This underscores the urgent need for further high-quality research to validate current clinical practices and enhance evidence-based guidelines for PI prevention in ICU settings. Investing in this area is essential to provide more robust and effective tools to healthcare professionals, improving clinical outcomes and patient safety. Given the limitations of qualitative synthesis, our findings serve as a foundation for future systematic reviews and meta-analyses that could quantitatively assess the impact of specific recommendations in clinical practice.

## Figures and Tables

**Table 1:** Search expressions in the CINAHL database.

Data Bases	Search Equation (February 2025)
CINAHL	(MH* “Risk Assessment”) OR (MH “Pressure Ulcer Prevention (Iowa NIC)”) OR TX** “Prevention” AND TX “Recommendations” OR TX “Good practice statements” OR TX “Considerations” OR (MH “Nursing Interventions”) OR “Interventions” OR TX “Care Bundles” OR (MH “Practice Guidelines”) OR (MH “Protocols”) OR TX “clinical guidelines” AND (MM*** “Pressure Ulcer”) OR (MH “Wounds and Injuries”) OR TX “Decubitus ulcer” OR TX “Bedsore” AND (MH “Intensive Care Units”) OR (MH “Critical Care”)

Note: MH*: exact subject headings; TX**: all text; MM***: exact major subject headings.

**Table 2:** Characteristics of studies according to year/country of publication, article title, study design, aims, and key conclusions.

Year/Country	Title	Study Design	Aims	Key Conclusions
2022/Turkey	Effectiveness of a PI* Prevention Care Bundle; Prospective Interventional Study in Intensive Care Units [15]	Prospective interventional study	Assess the effectiveness of a PI prevention care bundle	A PI prevention bundle was implemented in an intensive care unit, resulting in a decline in stage 1 pressure injuries
2022/UK**	Device-related pressure ulcers: SECURE prevention. Second edition [16]	Report	Address the need for greater recognition of medical-device-related pressure injuries (MDRPIs) and their causes, management, and prevention	Not applicable
2022/Italy	The prevention of PIs in the positioning and mobilization of patients in the ICU***: a good clinical practice document by the Italian Society of Anesthesia, Analgesia, Resuscitation and Intensive Care (SIAARTI) [17]	Observational study; multidisciplinary panel of experts	Support clinical decision-making concerning positioning and mobilization of the critically ill patient	A multidisciplinary panel of experts provided a list of good clinical practice principles based on available evidence. The statements may represent practical guidance for a broad range of professionals involved in the management of critically ill patients
2021/Saudi Arabia	The Effectiveness of the SKINCARE Bundle in Preventing MDRPI^+^ in Critical Care Units: A Clinical Trial [18]	Prospective, single-arm, open-label design	Examine the impact of a MDRPI prevention bundle on the incidence of acquired MDRPIs in critically ill patients	The SKINCARE bundle demonstrates the significant improvement of skin care through decreased cumulative incidence of acquired MDRPIs
2021/UK	Evaluating the impact on hospital acquired PI/ ulcer incidence in a United Kingdom NHS Acute Trust from use of sub-epidermal scanning technology [19]	Pragmatic study with SQUIRE guidelines	Measure the impact of adding scanning technology to the prevailing standard of care pathway on the incidence of category 2–4 PIs	Implementation of scanning technology into routine clinical practice achieves consistent reductions in the incidence of PIs
2021/Indonesia	Effects and interventions of PI prevention bundles of care in critically ill patients: A systematic review [20]	Systematic Review	Review the effects of PI prevention bundles of care on the incidence of PIs in critically ill patients.	PI prevention bundles of care in critically ill patients significantly reduced the incidence of PIs
2021/Brazil	Bundle For The Prevention Of PI Related To Medical Devices In Critically Ill Patients [21]	Observational Study	Develop and validate a set of measures for the prevention of MDRPIs in critically ill adult patients	The bundle created is valid and can be used as a care guide to facilitate activities and decisions regarding patients
2020/Brazil	MDRPI On Adults: An Integrative Review [22]	Integrative review	Identify factors associated with MDRPIs	There are several risk factors for the development of MDRPIs, which include severity of the patient, length of stay, humidity, skin friction, age, and use of vasoactive drugs and sedatives, among others
2020/Thailand	Development and validation of CAVE score in predicting presence of pressure ulcer in intensive care patients [23]	Prospective study	Develop and validate a PI risk assessment tool with good diagnostic properties in intensive care units	The predictive validity of the CAVE score is limited but comparable to the existing tools in Thailand. However, it has good diagnostic properties in young patients
2019/International	Prevention and Treatment of Pressure Ulcers/Injuries: Clinical Practice Guideline The International Guideline [11]	Report	Comprehensive analysis and assessment of the evidence available relating to the assessment, diagnosis, prevention, and treatment of PIs	Not applicable
2018/Brazil	PI Risk Prediction In Critical Care Patients: An Integrative Review [24]	Integrative review	Identify instruments used to assess PI risk in adult critically ill patients in an intensive care unit and analyse their predictive capacity	The present review showed a range of predictive, generic, and specific scales used for PI risk assessment in intensive care unit patients
2017/Ireland	Accuracy of ultrasound, thermography and subepidermal moisture in predicting pressure ulcers: a systematic review [25]	Systematic review	Establish the clinical significance, accuracy, and recommendations of ultrasound, thermography, photography, and subepidermal moisture measurements	SEM and ultrasound are promising in the detection and prediction of early tissue damage and PI presence. However, these methods should be further studied to clarify their potential for use more widely in PI prevention strategies
2015/Australia	Reducing PIs In Critically Ill Patients By Using A Patient Skin Integrity Care Bundle (Inspire) [26]	Before and after design	Test a patient skin integrity bundle, the InSPiRE protocol, for reducing PIs in critically ill patients	The intervention group, receiving the InSPiRE protocol, had a lower cumulative incidence of PIs, and fewer and less severe PIs developed over time
2014/USA^++^	Body Mass Index And Pressure Ulcers: Improved Predictability Of Pressure Ulcers In Intensive Care Patients [27]	Retrospective cohort study	Determine whether inclusion of body mass index enhanced use of the Braden scale in the prediction of PIs	Body mass index and incidence of PIs were related in intensive care patients. The addition of body mass index did not appreciably improve the accuracy of the Braden scale for predicting PIs
2014/UK	Pressure ulcers: prevention and management [12]	Report	Comprehensive analysis of the best evidence available relating to the prevention and management of PIs	Not applicable

Note: PI* = pressure injury; UK** = United Kingdom; ICU*** = intensive care units; MDRPI^+^ = medical-device-related pressure injury; USA^++^ = United States of America.

**Table 3:** Recommendations and statements of good practice; strengths of evidence and recommendations (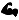).

Categories	Recommendations/Statements of Good Practice 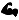 Strength of Evidence/Strength of Recommendations
Risk assessment instruments [11,12,15,16,17,20,24]	- Use a validated PI* risk assessment instrument that is sufficiently specific for the ICU** context, with assessment of additional risk factors [11,12,15,17]. 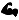 GPS***. - Generalists: Braden scale [11,17,20,24]; Braden ALB [17]; Norton scale [17]. 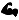 not available (n/a). - Specific: CALCULATE Scale [16,17]; Cubbin–Jackson scale [11,17,20,24]; COMHON index [11,17]; EVARUCI scale [11,17,20,24]; Suriadi and Sanada scale [11,20,24]. 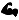 n/a.
Skin assessment [11,12,15,16,19,25,26]	- Perform a comprehensive and rigorous assessment of the skin and tissues from head to toe [11,12,15,26] with particular focus on the skin overlying bony prominences, including the sacrum, heels, hips, trochanters, pubis, thighs, and trunk [11,26]. 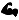 GPS/expert opinion.
	InSPiRE intervention bundle [26] The InSPiRE intervention is a bundle of PI prevention interventions that is based on the best available evidence, including international guidelines and the Australian Wound Management Association’s framework for PI prevention [26]. 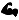 n/a.
	Technological alternatives for assessing the skin - Sub-Epidermal Moister (SEM) Scanner [11,16,19,25]. 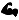 B2/↔. - Thermography [25]. 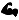 n/a. - Ultrasound [25]. 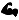 n/a.
Medical device surveillance [11,16,18,19,21,22]	- Assessment of the skin around/beneath medical devices every 12 h [11,15,16,21,22]. The assessment is determined based on clinical judgment of the patient’s condition and the level of risk associated with the device [16]. 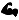 Expert opinion.
	SKINCARE Intervention Bundle: [18] - Bundle of interventions designed to prevent medical-device-related PI [18]. 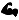 n/a.
Other alternatives for risk assessment [23,24,27]	- CAVE—cardiovascular–low albumin–ventilator–edema [23]. 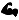 n/a. - Multi-pad pressure evaluator [24]. 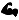 n/a. - Body mass index (BMI) [27]. 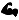 n/a.
Implementing best practices in clinical settings [11]	- At the organizational level, optimize the use of equipment and clinical decision support tools, and assess the knowledge healthcare professionals have about PIs as part of an education and quality improvement program to reduce the incidence of PIs [11]. 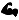 B1/↑↑.

Note: PI* = pressure injury; ICU** = intensive care unit; GPS*** = good practice statement.

## Data Availability

All the contributions presented in the study are included in the article; further inquiries can be directed to the corresponding author.

## Public Involvement Statement

There was no public involvement in any aspect of this research.

## Guidelines and Standards Statement

This manuscript was drafted against the methodology recommended by the Joanna Briggs Institute, adopting the Preferred Reporting Items for Systematic Reviews and Meta-Analyses Extension for Scoping Reviews (PRISMAScR) as a guide for writing the study [10].
